# Identification of candidate genes for salinity tolerance in *Japonica* rice at the seedling stage based on genome-wide association study and linkage mapping

**DOI:** 10.3389/fpls.2023.1184416

**Published:** 2023-05-10

**Authors:** Shanbin Xu, Jingnan Cui, Hu Cao, Shaoming Liang, Tianze Ma, Hualong Liu, Jingguo Wang, Luomiao Yang, Wei Xin, Yan Jia, Detang Zou, Hongliang Zheng

**Affiliations:** Key Laboratory of Germplasm Enhancement, Physiology and Ecology of Food Crops in Cold Region, Ministry of Education, Northeast Agricultural University, Harbin, China

**Keywords:** *Japonica* rice, GWAS, linkage mapping, salinity tolerance, seedling stage

## Abstract

**Background:**

Salinity tolerance plays a vital role in rice cultivation because the strength of salinity tolerance at the seedling stage directly affects seedling survival and final crop yield in saline soils. Here, we combined a genome-wide association study (GWAS) and linkage mapping to analyze the candidate intervals for salinity tolerance in Japonica rice at the seedling stage.

**Results:**

We used the Na+ concentration in shoots (SNC), K+ concentration in shoots (SKC), Na+/K+ ratio in shoots (SNK), and seedling survival rate (SSR) as indices to assess the salinity tolerance at the seedling stage in rice. The GWAS identified the lead SNP (Chr12_20864157), associated with an SNK, which the linkage mapping detected as being in qSK12. A 195-kb region on chromosome 12 was selected based on the overlapping regions in the GWAS and the linkage mapping. Based on haplotype analysis, qRT-PCR, and sequence analysis, we obtained LOC_Os12g34450 as a candidate gene.

**Conclusion:**

Based on these results, LOC_Os12g34450 was identified as a candidate gene contributing to salinity tolerance in Japonica rice. This study provides valuable guidance for plant breeders to improve the response of Japonica rice to salt stress.

## Introduction

Soil salinity is an important limiting factor affecting high and stable resistance in rice and expansion areas for the economically important crop (Ouhibi et al., 2014). Salt stress inhibits plant protein synthesis, reduces photosynthetic efficiency, causes ion imbalances and high osmotic stress, and leads to plant wilting and apoptosis (Ma et al., 2017). This stress response significantly reduces crop yield and has become an important environmental factor affecting crop growth and development. But at present, due to unreasonable irrigation methods and excessive fertilization, the salinization of soil is becoming more and more serious (Qadir et al., 2014). Rice can effectively help people by ensuring food security and advancing sustainable agriculture development. Growing rice on saline land can improve the utilization of saline soils and the soil conditions of the land. Rice is a moderately salt-sensitive crop, and its seedling salinity tolerance is a key factor determining its final yield. Therefore, its fine positioning can not only reveal its molecular mechanism, but also provide a theoretical basis for improving salt-tolerant varieties of rice.

Multiple rice genes control the complex quantitative trait known as salinity tolerance (Liang et al., 2015), and scientists have made a series of important research advances in cloning salt stress genes (Lin et al., 2004; Bimpong et al., 2014; Yu et al., 2017; Li et al., 2022). For example, *SKC1*, rice’s first salinity tolerance QTL, encodes a transporter protein of the HKT family. Previous studies have shown that *SKC1* protein is a sodium (Na^+^)-selective transporter protein that can effectively regulate the aboveground sodium and potassium (Na^+^/K^+^) balance and improve salinity tolerance (Ren et al., 2005). Meanwhile, Huang et al. (2009) reported a rice drought and salinity tolerance gene, *DST*, which negatively regulates salinity tolerance. The functional deletion of *DST* directly down-regulates the expression of genes related to hydrogen peroxide metabolism, reduces water evaporation under drought stress and Na^+^ entry into the plant, and ultimately improves the salinity tolerance of rice. Li et al. (2014) identified a lectin receptor-like kinase *SIT1* in rice, which mediates the salt-sensitive response in rice, and its rapid increase in response to high salt stimulation activates the downstream effectors *MAPK3* and *MAPK6*, which in turn increases ethylene content by activating ethylene synthase and causing an increase in reactive oxygen species, resulting in reduced survival under salt stress. Zhou et al. (2018) identified a receptor-like cytoplasmic kinase gene, *STRK1*, significantly improving salinity tolerance in rice. The *STRK1* protein undergoes autophosphorylation upon salt stress and significantly increases its activity by phosphorylating and activating CatC, thereby degrading the large amount of hydrogen peroxide produced by salt stress.

Cloning salinity tolerance genes has produced important breakthroughs in the molecular mechanism elucidation of important complex traits in rice, which have become a reference for studying the genetic mechanisms behind complex traits in rice. This work has significant application value and lays the foundation for identifying and cloning salinity tolerance genes in crops. Combining GWAS and QTL mapping for gene mining can significantly improve the efficiency of QTL identification, and the results can be verified against each other, resulting in stable and reliable QTLs. For example, Zhang et al. (2019) identified a candidate gene, *GmCDF1*, which is closely linked to soybean salinity tolerance on chromosome 8 through linkage and association analysis. Wu et al. (2016) combined GWAS and linkage analysis to identify 125 QTLs regulating maize male inflorescence size. Tang et al. (2021) used linkage localization and GWAS analysis to locate one QTL region controlling rice grain length, further identifying *OsGASR7* as a functional gene within this QTL interval. Therefore, combining the two methods is important for mining candidate genes for target traits.

This study explored the genetic mechanism of salinity tolerance as assessed by GWAS and linkage mapping using Na^+^ concentration in shoots (SNC), K^+^ concentration in shoots (SKC), Na^+^/K^+^ ratio in shoots (SNK), and seedling survival rate (SSR) at the rice seedling salt treatment. We identified Chr12_20864157 and *qSK12* on chromosome 12 by GWAS and linkage mapping, identifying an overlapping region of the 195-kb as a candidate region. Using haplotype analysis, qRT-PCR, and sequence analysis, *LOC_Os12g34450* was considered the most likely functional gene associated with salinity tolerance. The results procured by this study provide novel insights for improving salinity tolerance in *Japonica* rice.

## Materials and methods

### Plant materials

**Figure 1:**
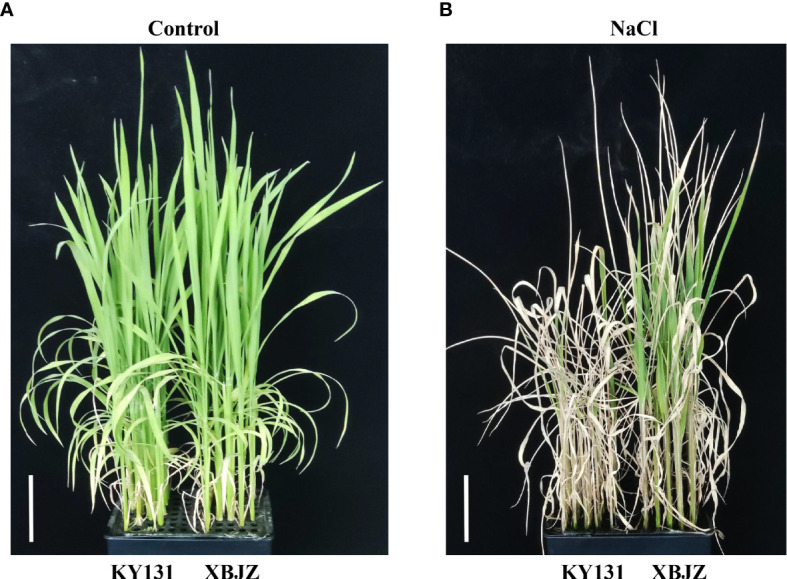
Phenotypes of KY131 and XBJZ seedlings under contral and salt stress. **(A)** Represent under control conditions. Bar = 5 cm. **(B)** Represent under salt stress conditions. Bar = 5 cm.

It consists of 295 *Japonica* rice varieties widely grown at home and abroad, of which domestic materials are mainly from Heilongjiang, Jilin, Liaoning, and Ningxia provinces, and foreign varieties were mainly from Korea, Russia, and Japan. The 195 RILs that comprised the linkage mapping population were a cross between the salt-sensitive Kongyu131 and the salt-tolerant Xiaobaijingzi. The salinity tolerance phenotypes of KY131 and XBJZ were shown in **Figure 1**. All rice varieties have been studied previously (Li et al., 2019; Li et al., 2020; Duan et al., 2022).

### Salinity tolerance evaluation at the seedling stage

The experiment was divided into two groups, named group T_1_ and group T_2_, and set up for three repetitions. In group T_1_, twenty-four uniformly germinated rice seeds of each variety were sown. One seed was placed in each hole and hydroponically grown with Yoshida nutrient solution. The germinated seeds were transferred to an artificial climatic chamber and incubated at 25 and 23°C with 14h light and 10h dark cycles, respectively. When seedlings grew to two leaves and one heart, salt stress treatment was performed with a pre-treatment sodium chloride (NaCl) concentration of 50 mmol/L.

After 3 days of pre-treatment, formal treatment was performed with a NaCl concentration of 120 mmol/L for 7 days. The shoot of each sample was dried at 120°C for 30 min and at 80°C to a constant weight. Weigh 0.1 grams (g) of the dry samples, add 5 ml of 1 mol/L HCl to the tube, and put it into a water bath for 6 h at 70°C in a constant temperature water bath shaker. The SNC and SKC of the samples were measured using a flame photometer (Sherwood 410, Cambridge, UK) and the SNK was calculated. In group T_2_, 100 uniformly germinated seeds of each variety were grown with Yoshida nutrient solution, with the same salt treatment as the T_1_ group. The culture medium was replaced every day, and replaced with the same medium as the control group after 7 days. After 10 days, the survival rate statistics counted the number of plants with new leaf production.

### GWAS for salinity tolerance

In total, 788,396 single nucleotide polymorphisms (SNPs) were used for genotyping 295 *Japonica* rice accessions for GWAS. The threshold for identifying significantly associated SNPs was set at –log_10_(*P*) > 5.26, according to a pre-laboratory study by Li et al. (2020). The Manhattan map was created using the R package ‘qqman’. Redundant SNPs with the smallest P values were filtered within a minimal distance interval and the LDBlockShow software was used to calculate the pairwise *R^2^* value between any two SNPs in the interval of leading SNPs ± 2 Mb. In the interval of 1.5-2.0 Mb of leading SNPs, the average of the top 10% *R^2^* values was recorded, plus 0.2 to define the LD attenuation interval of leading SNPs interval (Dong et al., 2021).

### QTL mapping for salinity tolerance

The genetic linkage map constructed using 195 RILs contained 527 bin markers (**Figure S1**). QTL localization was performed using the composite interval mapping method with QTL ICIMapping 4.2 software, and the threshold value was set to LOD>2.5, according to a pre-laboratory study by Li et al. (2020).

### Haplotype analysis of candidate gene

Non-synonymous mutant SNPs in the exonic regions of all genes in the candidate interval and SNPs in the promoter region (1.5 kb before ATG) were extracted from the RiceSNPSeekDatabase website (https://snp-seek.irri.org/_snp.zul) and haplotype analysis was performed using DnaSP software. Also, materials with different haplotypes needed to be greater than or equal to 10.

### Identification of candidate genes by gene expression and sequence analysis

The expression levels of the four genes of KY131 and XBJZ were verified by qRT-PCR analysis under salinated and normal conditions. qRT-PCR analysis was performed using Roche LightCycler96. All primer sequences were shown in **Table S3**. The CDS and promoter regions of KY131 and XBJZ candidate genes were cloned using PCR. Sequences comparison were performed using DNAMAN.

## Results

### Phenotypic variation

**Figure 2:**
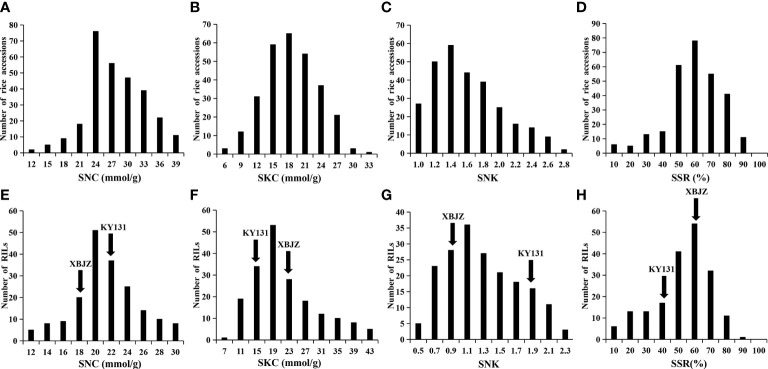
Phenotypic variation in the SNC, SKC, SNK, and SSR in 295 *Japonica* rice accessions and RIL lines. **(A–D)** Represent the SNC, SKC, SNK, and SSR of 295 rice accessions. **(E–H)** Represent the SNC, SKC, SNK, and SSR of RIL lines.

In this study, we analyzed the phenotypes of 295 *Japonica* rice accessions and RIL lines at the seedling stage under salinity stress and evaluated four salinity tolerance indices: SNC, SKC, SNK, and SSR. Under the salinity stress treatment, the SNC, SKC, SNK, and SSR among the 295 rice accessions varied in range from 10.14–38.83 mmol/g, 5.21–32.82 mmol/g, 0.79–2.78, and 9.33–88.66%, respectively, and the coefficient of variation was 20.13%, 26.73%, 28.61%, and 28.12%, respectively (**Table S1**). The variation for SNC, SKC, SNK, and SSR in the RIL lines ranged from 8.12–29.56 mmol/g, 6.98–39.56 mmol/g, 0.46–2.19, and 8.66–89.33%, respectively, and the coefficient of variation was 20.48%, 40.58%, 37.01%, and 32.99%, respectively (**Table S1**). Phenotypic data indicate that this study’s natural and RIL populations have abundant phenotypic variation in rice seedling salinity tolerance. Meanwhile, the values of these four traits differed significantly between the two parents (**Figures 2E–H**; **Table S1**), the phenotype values of the four traits indicating that XBJZ was more salinity-tolerant than KY131. The SNC, SKC, SNK, and SSR phenotypic values in the 295 rice accessions and RIL lines were normally distributed, demonstrating that these indices are quantitative traits under the control of numerous factors (**Figure 2**).

### GWAS for salinity tolerance-related traits in a natural population

**Figure 3:**
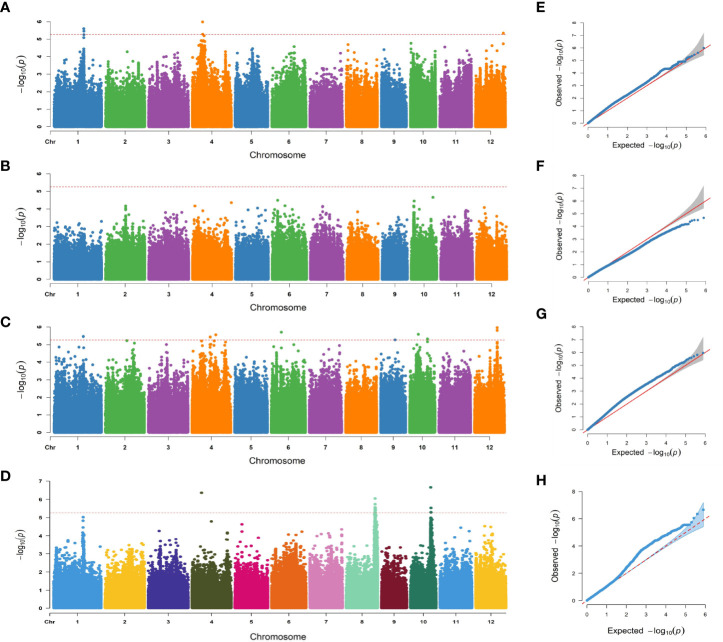
Manhattan plots and quantile-quantile (Q-Q) plots of GWAS for the SNC, SKC, SNK, and SSR. **(A–D)** Manhattan plot for the SNC, SKC, SNK, and SSR. **(E–H)** Q-Q plot for the SNC, SKC, SNK, and SSR.

The 788,396 SNPs obtained from previous studies were used for GWAS analysis (Li et al., 2019; Zheng et al., 2022). Manhattan and quantile–quantile plots were shown in **Figure 3**. Fourteen lead SNPs significantly associated with SNC, SNK, and SSR were provided in **Table 1**. Three QTLs associated with SNC were detected and located on chromosomes 1, 4, and 12, with *R^2^* values ranging from 9.20–10.36%. No QTL associated with SKC was detected, and eight QTLs associated with SNK were detected and located on chromosomes 1, 4, 6, 9, 10, and 12, with *R^2^* values ranging from 9.05–10.30%. Meanwhile, three QTLs associated with SSR were detected and located on chromosomes 4, 8, and 10, with *R^2^* values ranging from 9.76–12.02% (**Table 1**).

**Table 1:** Lead SNPs for SNC, SNK, and SSR identified by GWAS.

Traits	Lead SNP	Chromosome	Position	P value	*R* ^2^(%)	Known QTLs	Known genes
SNC	Chr1:27088613	1	27088613	2.48E-06	9.66	*qNAUP-1a* (Sabouri and Sabouri, 2008)	
	Chr4:9305690	4	9305690	1.02E-06	10.36	*qPn4b* (Tong et al., 2006)	
	Chr12:25487805	12	25487805	4.49E-06	9.20		
SNK	Chr1:26805021	1	26805021	3.44E-06	9.36	*qNAUP-1a* (Sabouri and Sabouri, 2008)	*OSBZ8* (Mukherjee et al., 2006)
	Chr4:16679366	4	16679366	3.70E-06	9.46	*qPn4b* (Tong et al., 2006)	
	Chr4:21840798	4	21840798	2.77E-06	9.57	*qDTF4.1s* (Mohammadi et al., 2013)	*OsCLC-1* (Nakamura et al., 2006)
	Chr6:9215769	6	9215769	1.99E-06	9.83		
	Chr9:13269677	9	13269677	5.31E-06	9.05		
	Chr10:7916859	10	7916859	2.61E-06	9.78	*SalTol10-1* (Islam et al., 2011)	
	Chr10:16340314	10	16340314	4.70E-06	9.32	*SalTol*10-1 (Islam et al., 2011)	
	Chr12:20864157	12	20864157	1.10E-06	10.30	*QSst12* (Cheng et al., 2012)	
SSR	Chr4:8601162	4	8601162	4.40E-07	9.76	*qPn4b* (Tong et al., 2006)	
	Chr8:26210079	8	26210079	8.94E-07	10.68	*qGY8.1s* (Mohammadi et al., 2013)	
	Chr10:18354286	10	18354286	2.20E-07	12.02	*qSKC-10b* (Ghomi et al., 2013)	

R^2^ (%): Phenotypic variance explained.

### Linkage mapping for salinity tolerance at the seedling stage

**Figure 4:**
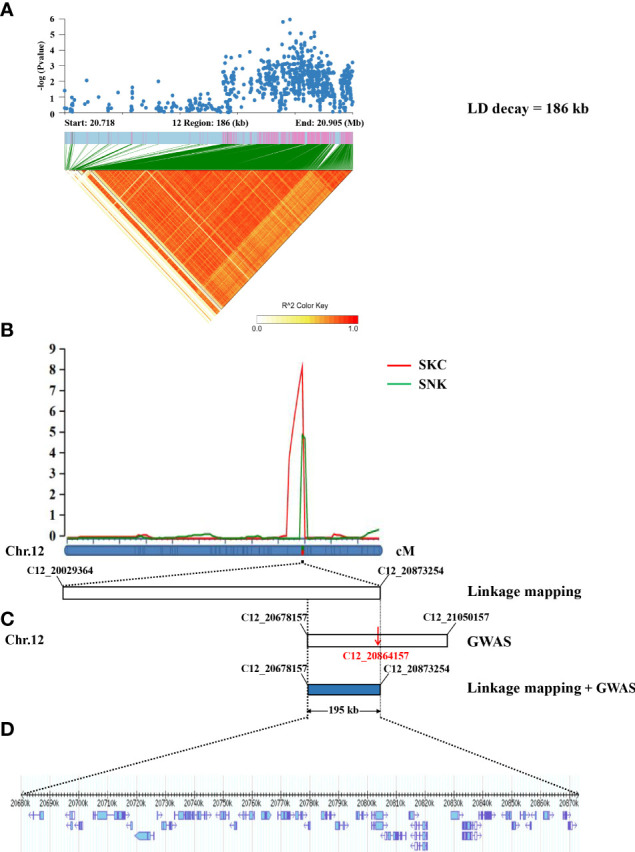
Identification of candidate genes by GWAS and linkage mapping. **(A)** The local Manhattan plot (top) and LD heatmap (bottom) surround the lead SNP. **(B)** Salinity tolerance-related QTLs were identified in 195 RILs and mapped to the interval between markers C12_20029364 and C12_20873254 by linkage mapping. **(C)** The physical location of the lead SNP (C12_20864157) on chromosome 12 was detected by the GWAS (LD decay = 186 kb). **(D)** The 195-kb region contained 35 genes.

A total of five QTLs associated with SNC, SKC, SNK, and SSR were identified on chromosomes 1, 4, and 12 using linkage mapping (**Table 2**; **Figure S1**), with LOD values from 2.51–8.22 and proportions of phenotypic variation ranged from 5.49–18.27%. In addition, *qSKC12* and *qSNK12* were considered to be the same QTL because of the same interval, named *qSK12* (chromosome 12) (**Figure 4B**), located in the physical region between markers C12_20029364 and C12_20873254 and explaining 11.54–18.27% of the phenotypic variation. The GWAS identified the lead SNPs, Chr12_20864157, associated with an SNK, which the linkage mapping detected as being in *qSK12*. The LD block region on chromosome 12 was predicted to be 20.718–20.905 Mb (186 kb) (**Figure 4A**). A 195-kb overlap region was filtered out based on the GWAS and the linkage mapping (**Figure 4C**).

**Table 2:** QTLs for SNC, SKC, SNK, and SSR identified by linkage mapping analysis in 195 RILs.

Traits	QTLs	Left Marker	Right Marker	Chr.	LOD	*R^2^* (%)	Additive effect	Known QTLs	Known genes
SNC	*qSNC12*	C12_23512814	C12_24090250	12	2.51	6.17	1.03	*qSH12.1* (Wang et al., 2012)	*OsMYB91* (Zhu et al., 2015)
SKC	*qSKC12*	C12_20029364	C12_20873254	12	8.22	18.27	-3.50	*QSst12* (Cheng et al., 2012)	
SNK	*qSNK12*	C12_20029364	C12_20873254	12	4.99	11.54	-0.15	*QSst12* (Cheng et al., 2012)	
SSR	*qSSR1*	C1_33960158	C1_34422650	1	3.18	5.49	-0.05	*qPH1.1s* (Mohammadi et al., 2013)	
	*qSSR4*	C4_22587609	C4_25541936	4	4.28	8.88	0.06	*qDTF4.1s* (Mohammadi et al., 2013)	*OsNAC2* (Shen et al., 2017)

R^2^ (%): Phenotypic variance explained.

### Haplotype analysis of candidate genes

**Figure 5:**
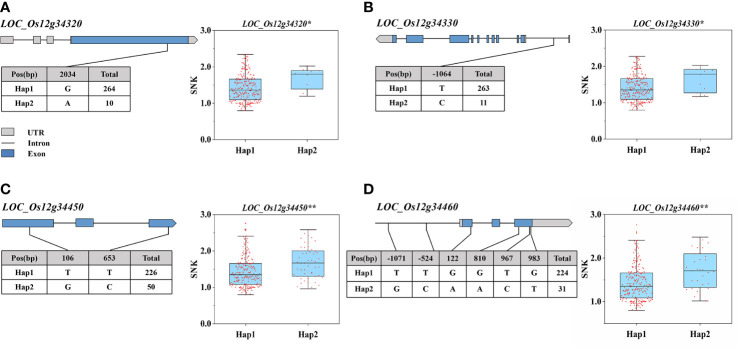
Haplotype analysis of *LOC_Os12g34320*, *LOC_Os12g34330*, *LOC_Os12g34450*, *LOC_Os12g34460*. **(A–D)** Represent the gene structure and haplotype analysis of *LOC_Os12g34320*, *LOC_Os12g34330*, *LOC_Os12g34450*, *LOC_Os12g34460*. (The * and ** suggest significance of ANOVA at P < 0.05 and P < 0.01, respectively).

According to the *Phytozome* database, the 195-kb candidate interval did not include known salinity tolerance genes from previous studies. The 195-kb region on chromosome 12 contained 35 genes (**Figure 4D**; **Table S2**). We performed haplotype analysis of 35 genes and found that 4 genes (*LOC_Os12g34320*, *LOC_Os12g34330*, *LOC_Os12g34450*, and *LOC_Os12g34460*) within the overlapping interval were associated with significantly different haplotypes of SNKs. *LOC_Os12g34320* and *LOC_Os12g34450* were classified into two haplotypes by non-synonymous mutant SNPs in the exon region. One SNP located in the 5′ untranslated region of *LOC_Os12g34330* formed two haplotypes, while no non-synonymous SNP was found in the exon region. The haplotype analysis of *LOC_Os12g34460* indicated that 2 SNPs were identified in the promoter region and 4 non-synonymous SNPs were detected in the exon region (**Figures 5A–D**).

### Identification of candidate genes by gene expression and sequence analysis

**Figure 6:**
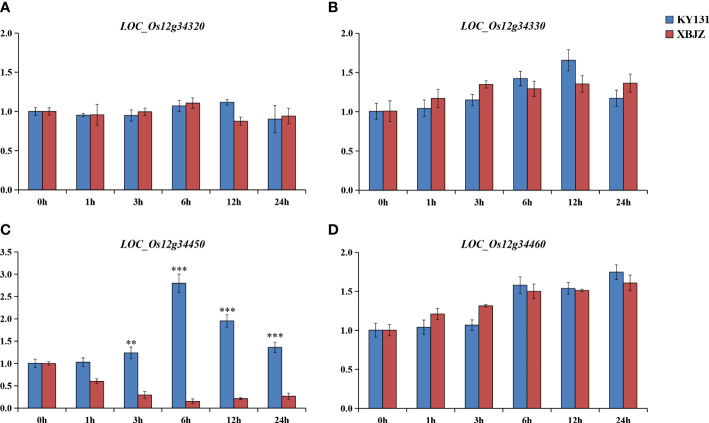
Expression patterns of the four genes under normal growth conditions and salinity stress. **(A–D)** represent the gene expression of LOC_Os12g34320, LOC_Os12g34330, LOC_Os12g34450, LOC_Os12g3446 under normal growth conditions and salinity stress. (**P < 0.01, ***P < 0.001, Students’t-test).

The two parents, KY131 and XBJZ, were treated with 120 mM NaCl for 0, 1, 3, 6, 12, and 24 h, respectively. The expression patterns of these four genes were assessed by qRT-PCR analysis. The mean results in triplicate were shown in **Figure 6**. Among these four genes, salinity stress did not increase the expression levels of *LOC_Os12g34320*, *LOC_Os12g34330*, or *LOC_Os12g34460* (**Figures 6A, B, D**). *LOC_Os12g34450* was significantly induced by salinity stress, and the expression levels were significantly different between KY131 and XBJZ, which showed opposite expression patterns. The expression level of *LOC_Os12g34450* was more than 18-fold higher in KY131 than in XBJZ under 6 h of salinity stress (**Figure 6C**). Meanwhile, we performed qRT-PCR analysis of other functionally annotated genes within the 195-kb region, and the results of the four genes expressed between the two parents are shown in **Figure S2**, which were not differentially expressed between the two parents. After observing these results, we completed further sequencing of the promoter regions and genes of *LOC_O12g34450* in the two parents KY131 and XBJZ. Compared with the sequence of KY131, *LOC_Os12g34450* of XBJZ had a 2-bp deletion (A and C bases) and 2 SNPs (T→G, G→A) in the first exon of the CDS region, 1 SNP (T→C) in the third exon, and multiple SNPs in the promoter region containing 1 cis-element (TGA-element) related to auxin response (AACGAC→GACGAT) (**Figure S3**). Therefore, *LOC_Os12g34450* was considered a functional gene associated with salinity tolerance. *LOC_Os12g34450* encodes an auxin-binding protein 4 precursor gene, which has not been reported to affect salinity tolerance in rice in previous studies. In subsequent studies, transgenic plants will be examined for the biological function of their associated genomic variant’s responses to salinity tolerance in *Japonica* rice.

## Discussion

Rice seedlings have poor salinity tolerance and are susceptible to salt stress, which seriously affects their planting and yield. Therefore, cultivating salinity tolerance in seedlings has great practical significance for agricultural production (Nam et al., 2015). This study selected SNC, SKC, SNK, and SSR as indicators to assess salinity tolerance in rice seedlings, which have also been used in previous studies (Rahman et al., 2016). Under salinity stress conditions, the Na^+^ content in rice seedling shoots and roots increases, and the K^+^ content decreases. This chemical change leads to a higher Na^+^/K^+^ ratio and disrupts the ionic balance by decreasing Mg, Zn, and Mn content (Tuncturk et al., 2008), also leading to osmotic stress and growth inhibition (Munns, 2011).

Salinity tolerance is a complex trait, and identifying such QTLs helps obtain relative salt tolerance genes for molecular-assisted breeding (Ganie et al., 2019). Eleven and three QTLs were identified by GWAS and linkage mapping, respectively, and these were close to or overlapped with the loci of some known genes and QTLs compared with the results of previous studies. For example, *OsCLC-1*, which encodes a voltage-gated chloride channel protein, could avoid ion toxicity by transporting chloride ions across the vesicle membrane to the vesicles (Nakamura et al., 2006); the lead SNP Chr4_21840798 identified by GWAS was approximately 45 kb closer to *OsCLC-1*. *OsMYB9*, encoding an R2R3-type MYB transcription factor that functions in the ABA-mediated signaling pathway, thereby improving the salinity tolerance of rice (Zhu et al., 2015), was within the *qSNC12* identified by the linkage mapping. Additionally, Shen et al. (2017) found that *OsNAC2*, was able to regulate abiotic stress response, *OsNAC2* overexpression plants showed reduced tolerance under salt stress, and *OsNAC2* was within the *qSSR4* identified by the linkage mapping.

Sabouri and Sabouri (2008) detected a salinity tolerance-associated QTL (*qNAUP-1a*) on chromosome 1, and the lead SNPs (Chr1_27088613 and Chr1_26805021), detected by GWAS, were located within *qNAUP-1a*. Islam et al. (2011) detected a salinity tolerance-related QTL (*SalTol10-1*) on chromosome 10, and the lead SNPs Chr10_7916859 and Chr10_16340314 detected by GWAS were both located within *SalTol10-1*. In addition, linkage mapping revealed that the QTL *qSNC12* was within the salinity tolerance interval *qSH12.1* (Wang et al., 2012), which supported our findings. In our study, GWAS and linkage mapping identified another salinity tolerance QTL, *QSst12*, containing Chr12_20864157 and *qSK12*.

The *LOC_Os12g34450* was considered the most likely functional gene associated with salinity tolerance. The expression of *LOC_Os12g34450* was significantly up-regulated in XBJZ and down-regulated in KY131 after salinity stress. Meanwhile, we found that *LOC_Os12g34450* of XBJZ contained multiple SNPs in the promoter region, including a cis-element (TGA-element) related to auxin response (AACGAC→GACGAT), three non-synonymous mutation SNPs (T→G, G→A, T→C) in the CDS region, and a 2-bp deletion (A and C bases) in the first exon compared with the sequence of KY131. These SNPs and deletions result in a frameshift that leads to a premature stop codon, which leads to premature termination of the mRNA of the *LOC_Os12g34450*. *LOC_Os12g34450* is predicted to encode an auxin-binding protein, and accumulating evidence in recent years supports an important role for auxin in abiotic stress responses in plants.

In *Arabidopsis*, the *NTM2* plays an important role in regulating plant seed germination under high-salt stress (Jung and Park, 2011). Overexpression of *OsmiR393* in rice resulted in the down-regulation of two auxin receptor gene homologs (*OsTIR1* and *OsAFB2*), which reduced salinity tolerance in rice (Xia et al., 2012). Deng et al. (2022) found that *RST1* encodes the growth factor response factor *OsARF18* and that *RST1* loss of function leads to up-regulation of *OsAS1* expression, which improves nitrogen utilization, reduces Na^+^/K^+^ ratios, and decreases NH_4_^+^ overaccumulation by promoting asparagine synthesis, thereby improving salt tolerance and yield of plants.

## Data availability statement

The original contributions presented in the study are included in the article/**Supplementary Material**, further inquiries can be directed to the corresponding authors.

## Author contributions

SX, DZ, and HZ designed the study and provided experimental materials. SX, JC, HC, SL, and TM performed the experiments. SX, JC, and HZ analyzed the results and wrote the manuscript. All authors contributed to the article and approved the submitted version.

## Supplementary material

Supplementary Figure 1 Genetic linkage map and QTL mapping results.

Supplementary Figure 2 Expression patterns of the other four genes under normal growth conditions and salinity stress (** P < 0.01, *** P < 0.001, Students’t-test).

Supplementary Figure 3 The gene structure and sequence difference analysis of *LOC_Os12g34450*. **(A)** The gene structure of *LOC_Os12g34450*. **(B)** The sequence difference analysis of *LOC_Os12g34450* in Xiaobaijingzi, Kongyu131, and Nipponbare.
