# Supplementary figures and images for: Identification of candidate genes for salinity tolerance in Japonica rice at the seedling stage based on genome-wide association study and linkage mapping
Source: Front Plant Sci. 2023 May 10;14:1184416. doi: 10.3389/fpls.2023.1184416 (PMC10206223; doi:10.3389/fpls.2023.1184416)

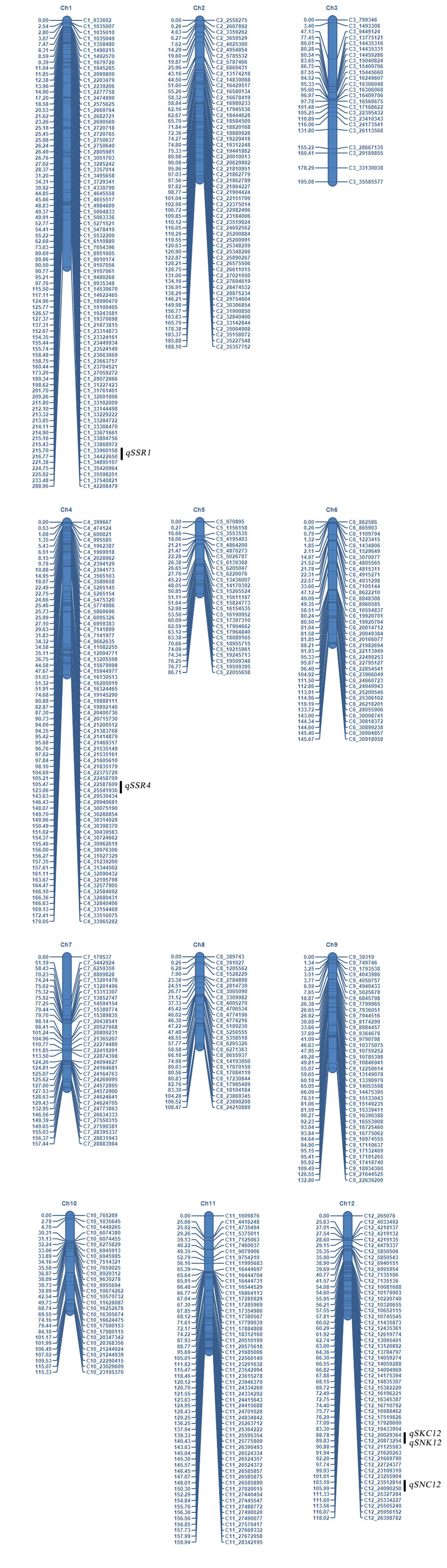

Supplement: Supplementary Figure 1 — Genetic linkage map and QTL mapping results. [file Image_1.tif]

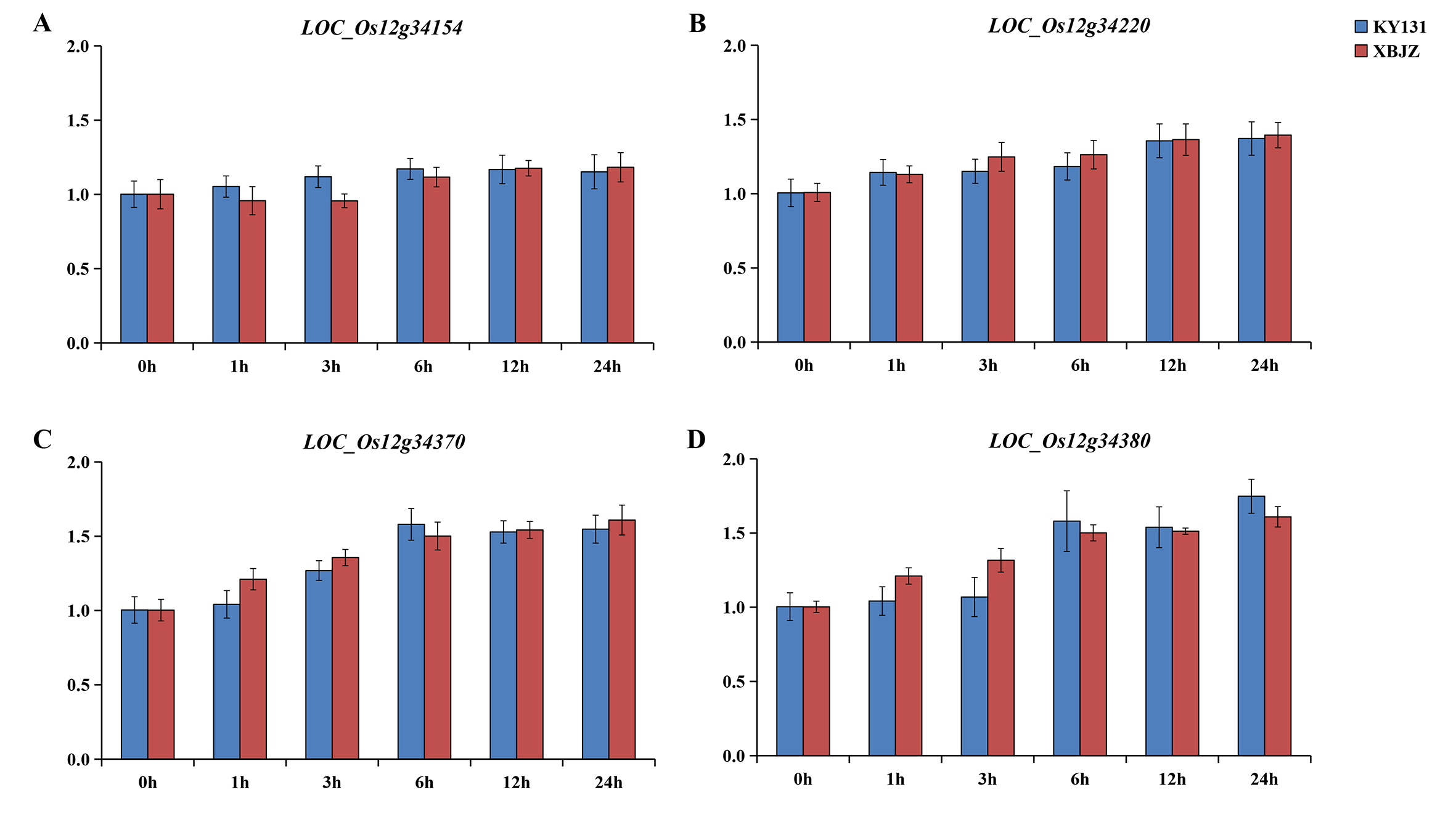

Supplement: Supplementary Figure 2 — Expression patterns of the other four genes under normal growth conditions and salinity stress (** P < 0.01, *** P < 0.001, Students’t-test). [file Image_2.tif]

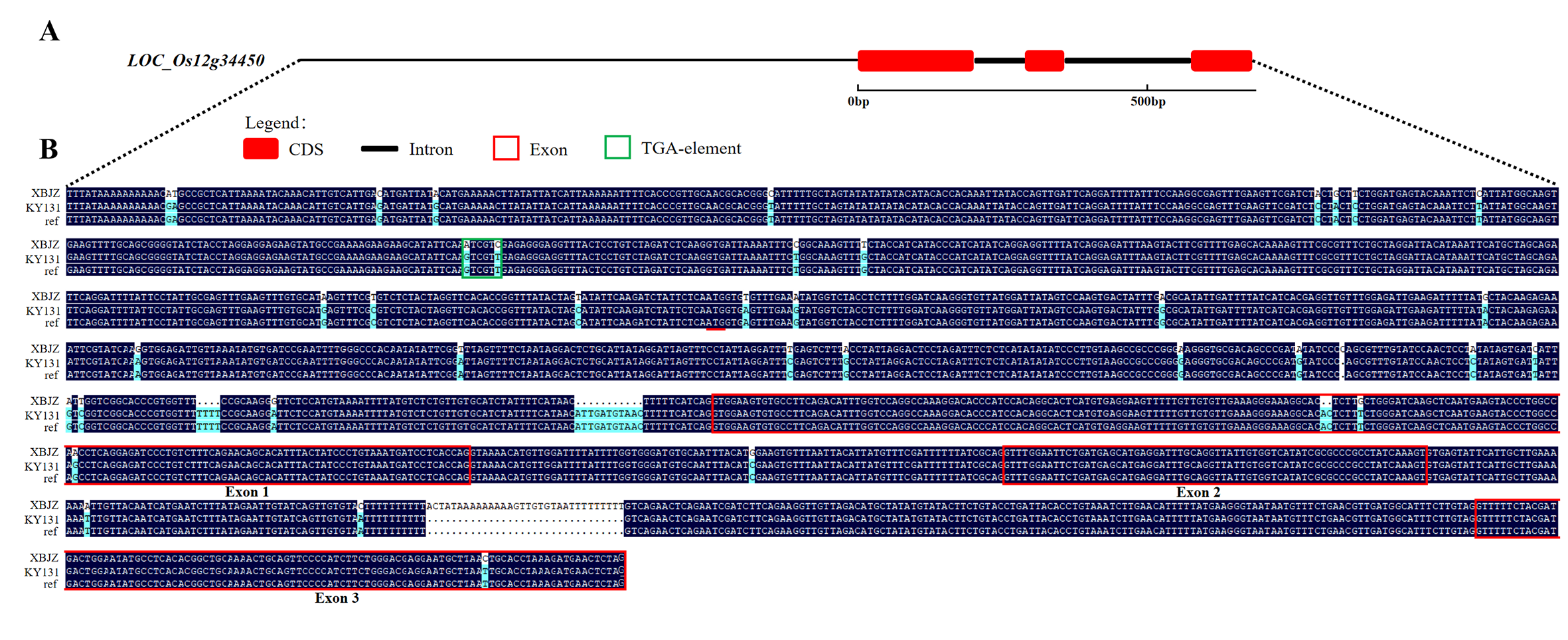

Supplement: Supplementary Figure 3 — The gene structure and sequence difference analysis of LOC_Os12g34450. (A) The gene structure of LOC_Os12g34450. (B) The sequence difference analysis of LOC_Os12g34450 in Xiaobaijingzi, Kongyu131, and Nipponbare. [file Image_3.tif]
